# Nondestructive detection of apple crispness via optical fiber spectroscopy based on effective wavelengths

**DOI:** 10.1002/fsn3.1222

**Published:** 2019-10-03

**Authors:** Fupeng Ni, Xiaowen Zhu, Fang Gu, Yaohua Hu

**Affiliations:** ^1^ College of Mechanical and Electronic Engineering Northwest A&F University Yangling China; ^2^ Key Laboratory of Agricultural Internet of Things Ministry of Agriculture Yangling China; ^3^ Shaanxi Key Laboratory of Agricultural Information Perception and Intelligent Service Yangling China

**Keywords:** apple crispness, artificial neural network, effective wavelengths, optical fiber spectroscopy, partial least squares method, successive projections algorithm

## Abstract

Crispness is regarded as a significant quality index for apples. Currently, destructive sensory evaluation is the accepted method used to detect apple crispness, making it essential to develop a method that can detect apple crispness in a nondestructive manner. In this study, spectroscopy was proposed as the nondestructive technique for detecting apples' crispness, ultimately obtaining a spectral reflectance curve between 450 nm and 1,000 nm. In order to simplify and improve modeling efficiency, successive projections algorithm (SPA) and x‐loading weights (x‐LW) methods were used to select the most effective wavelengths. Partial least squares (PLS) algorithm, radial basis neural networks (RBNN), and multilayer perceptron neural networks (MLPNN) methods were used to establish the models and to predict the crispness of “Fuji” and “Qinguan” apple varieties. Based on the full wavelength (FW), the prediction accuracy of the PLS model for “Fuji” and “Qinguan” apple varieties was 92.05% and 95.87%, respectively. The effective wavelengths selected via SPA for the “Fuji” apple variety were 450.41 nm, 476.80 nm, 677.75 nm, and 750.72 nm, and the effective wavelengths selected via x‐LW for the “Qinguan” apple variety were 542.51 nm, 544.79 nm, 676.96 nm, and 718.29 nm. The prediction accuracy of the PLS model based on effective wavelengths for “Fuji” and “Qinguan” apple varieties reached 91.31% and 96.41%, respectively. Compared with the RBNN model, the MLPNN model achieved better prediction results for both “Fuji” and “Qinguan” apples, with the prediction accuracy reaching 97.8% and 99.9%, respectively. Based on the above findings, effective wavelength selection and MLPNN modeling were able to detect apple crispness with the highest accuracy. Overall, it can be concluded that the less effective wavelengths are conducive to developing an instrument for crispness detection.

## 1 INTRODUCTION

Apples are popular among consumers due to their high nutritional value and their ability to improve and beautify skin. China has the largest area of apple orchards and the highest total apple yield in the world, with the “Fuji” apple being the major Chinese cultivar, accounting for more than 70% of the apple acreage in China (Qiang, Bei‐Bei, Min‐Ji, Qin‐Ping, & Zhen‐Hai, 2018). The “Qinguan” apple is another important cultivar in China's apple industry, with its large size, red color, superior storability, and unique flavor making it popular with consumers. Crispness is a vitally significant factor when evaluating apple texture quality, and however, there are distinct differences between “Fuji” and “Qinguan” apple cultivars in this regards. Therefore, it is of great importance to study both the “Fuji” and “Qinguan” apple cultivars when determining an improved method for assessing crispness. Apple losing their crisp quality during storage leads to consumers' dissatisfaction, as such fruits no longer feel fresh during consumption, with the apple varieties that maintain crispness having a greater long‐term consumer appeal (Chang, Vickers, & Tong, 2018). Nowadays, destructive sensory evaluation is the major method used for detecting crispness, and destructive testing is wasteful and costly. Therefore, it is imperative to develop a nondestructive method. In recent years, spectroscopy has been proposed as a nondestructive crispness detection technique. Some studies have explored spectroscopy as a method for the nondestructive analysis of other crops and fruits, research which has included apples (Dong & Guo, 2015; Kong, Zhang, Huang, Liu, & He, 2018; Li et al., 2018; Peng et al., 2018; Sun, Künnemeyer, Mcglone, & Rowe, 2016). Spectroscopy techniques were also used in other fields such as oil and soil detection (Feng et al., 2018; He et al., 2018; Nie, Dong, He, & Xiao, 2018). Although some studies have investigated the use of spectroscopy for the quality evaluation of apples (Betemps et al., 2012; Guang‐Hui, Ren, Ren, & Zhao, 2012), and some studies have explored crispness detection using contact acoustic emission (Belie, Smedt, & Baerdemaeker, 2000; Demattè et al., 2014; Piazza & Giovenzana, 2015; Zdunek, Konopacka, & Jesionkowska, 2010), there has been little research done on the crispness of “Fuji” and “Qinguan” apple varieties specifically, and a nondestructive crispness detection method, combining spectroscopic and chemometric approaches, remains unexplored. In order to obtain an accurate and stable prediction model based on the spectroscopic technique, different models were developed via a range of modeling methods, including partial least squares (PLS) and artificial neural networks (ANN) (Cliff & Bejaei, 2018; Feng et al., 2018; He, Jian, Ying, & Li, 2015). However, these modeling methods were not applied to the detection of apple crispness via optical fiber spectroscopy.

The specific goals of this study were: (a) to determine effective wavelengths for assessing the apples' crispness; (b) to establish quantitative prediction models for apple crispness based on the effective wavelengths; and (c) to validate and optimize the prediction models for apple crispness.

## 2 MATERIALS AND METHODS

### 2.1 Sample preparation

“Fuji” and “Qinguan” apples are two the main cultivars in China, and however, the crispness of “Fuji” and “Qinguan” is distinctly different, with the “Fuji” apple tending to be far crisper than “Qinguan.” Therefore, this study was conducted using both the “Fuji” and “Qinguan” apple cultivars, which were obtained in October 2018 from Baishui County, Shaanxi Province, China. To confirm that testing process took place under identical conditions, 60 “Fuji” apples and 60 “Qinguan” apples of a similar shape were placed in an Artificial Climate Chamber (PRx‐250B, Ningbo Saifu Experimental Instrument Co., Ltd.) where the temperature was set to 4 ± 2°C, and the relative moisture was set to a humidity level of 85 ± 1%. The step test method was adopted for the experimental scheme. Sixty “Fuji” apples and 60 “Qinguan” apples were marked, and then, each variety was divided into 12 groups on average. The experiment was conducted over a period of 12 weeks.

### 2.2 Equipment and methods for spectra acquisition

**Figure 1:**
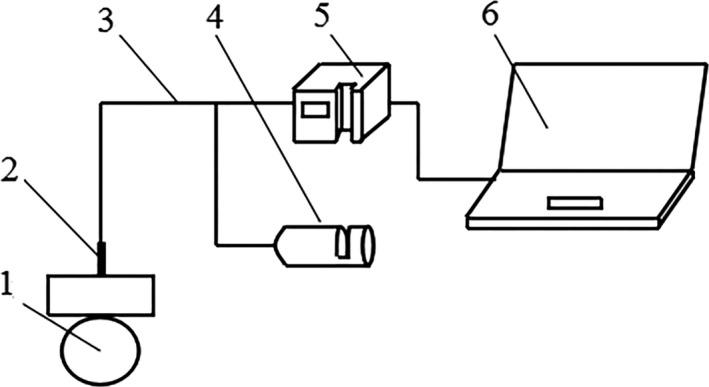
Diagrammatic structure of experiment. 1: apple; 2: Optical fiber probe; 3: Optical fiber; 4: Light source; 5: Spectrometer; 6: Computer that operated spectrometer

Spectra were measured using an optical fiber spectrometer (USB4000, Ocean optics) equipped with SpectraSuite (64 bit) software, an optical fiber (Ocean optics QR200‐7‐UV‐VIS), and an optical fiber probe (Ocean optics QR200‐7‐UV‐VIS). The spectrometer was preheated for 15 min before use. The wavelength range of the spectrometer is 350–1000 nm, the range of integration time is from 3.8 ms to 10 s, the optical resolution of the spectrometer is 1.5–2.3 nm FWHM, and the sampling interval is 0.21 nm. A white panel was used as a reference in order to eliminate interference by the light source itself. All spectra were obtained using an optical fiber probe. Before collecting the apple spectra, some parameters were set by the SpectraSuite software. The integration time was set to 10 ms, the scan‐to‐average was set to 15, and the boxcar smoothing was set to 10. Spectra were obtained every week from three points which were evenly distributed along the equator of each apple. Figure 1 depicts the physical structure of the spectrometer, as well as the diagrammatic structure of the spectra collection process.

### 2.3 Equipment and methods for crispness measurement

An Artificial Climate Chamber (PRx‐250B, Ningbo Saifu Experimental Instrument Co., Ltd. China) was used to store samples. The Texture Analyzer (TA.XT plus) was used to detect crispness values of the samples, and a computer (Lenovo QiTianM7300) was used to operate the Texture Analyzer software and store the detection information.

After the spectra were collected, samples were conveyed to detect crispness via a Texture Analyzer. Three cylindrical samples were evenly chosen from the equator of each apple at regular intervals which corresponded with the points where the spectra were collected. In addition, both the diameter of the cross section and length of each sample were 1 cm. Before the crispness was detected, the Texture Analyzer was set according to the desired requirements. The compression speed was set to 1 mm/s, the amount of compression was set to 80%, and the trigger force was set to 50 N (Liu et al., 2016).

### 2.4 Chemometrics methods

The partial least squares (PLS) method is a most widely used regression modeling method in spectral data analysis, favored for its flexibility and reliability when dealing with redundant spectral data. The versatility of PLS makes it possible to establish a regression model in a scenario where the number of samples is less than the number of variables (Lin et al., 2018). The PLS method includes several algorithms. The nonlinear iterative partial least squares (NIPLS) algorithm was chosen to establish a prediction model. This algorithm combines feature extraction with classifier design. When compared with traditional algorithms, such as Fisher discriminant analysis (FDA) and Bayes discriminant analysis (BDA), NIPLS is simple, robust, and capable of clear qualitative explanation, with the ability to apply this to the classification recognition. In addition, NIPLS is powerful in terms of multicollinearity, particularly when the number of predictor variables is large and the sample size is small, making it a novel and efficient algorithm for pattern recognition (Shi‐Fei, Zhong‐Zhi, & Feng‐Xiang, 2008).

The artificial neural network (ANN) method includes a back propagation neural network (BPNN), an evolutionary neural network (ENN), an extreme learning machine (ELM), a general regression neural network (GRNN), a radial basis neural network (RBNN) (Feng et al., 2018), and a multilayer perceptron neural network (MLPNN). Both MLPNN and RBNN were used to develop the models in this study.

### 2.5 Software and model evaluation methods

The data processing software included Unscrambler X 10.1 (Camo ProcessAS Oslo Norway), Microsoft Excel 2010, SPSS (Statistical Package for the Social Sciences, version 18), and Matrix Laboratory (MATLAB 8.5, R2015a, MathWorks company). Throughout the PLS modeling process using the software Unscrambler X 10.1, the modeling effect was evaluated by the determination coefficient (*R* ^2^) and the root mean square error (RMSE). The closer the *R* ^2^ is to 1, the closer the RMSE is to 0, the better the performance of the prediction model will be (Kawamura et al., 2017). The prediction residual error sum of squares (PRESS) and standard error of cross‐validation (SECV) obtained via cross‐validation were used to evaluate the models' whole performance. Throughout the PLS modeling process using the software SPSS, the determination coefficient (*R* ^2^), the sum of square deviation, and relative deviation were used to evaluate the ANN model; the lower the sum of square deviation and relative deviation are, the more effective the model will be.

### 2.6 Effective wavelength selection

The successive projections algorithm (SPA) provides a direct method to solve the collinearity problem (Li, Sun, Zhou, & He, 2015). The method starts at one wavelength and then calculates its projection at the unselected wavelength in each loop. The wavelength with the maximum projection vector is introduced into the wavelength combination (Li et al., 2018; Liu & Zhang, 2012).

The x‐loading weights (x‐LW) method is based on the PLS modeling results. The influence of each wavelength on the model's performance is evaluated using absolute values of load coefficients. The wavelength at the maximum absolute value of the load coefficient of each implicit variable is regarded as the most effective wavelength corresponding to that implicit variable. The number of effective wavelengths is the same as that of the implicit variable of the model (Cheng, Xie, Wang, Yong, & Shao, 2014; Pan et al., 2017).

## 3 RESULTS AND DISCUSSIONS

### 3.1 Spectral reflectance curves

**Figure 2:**
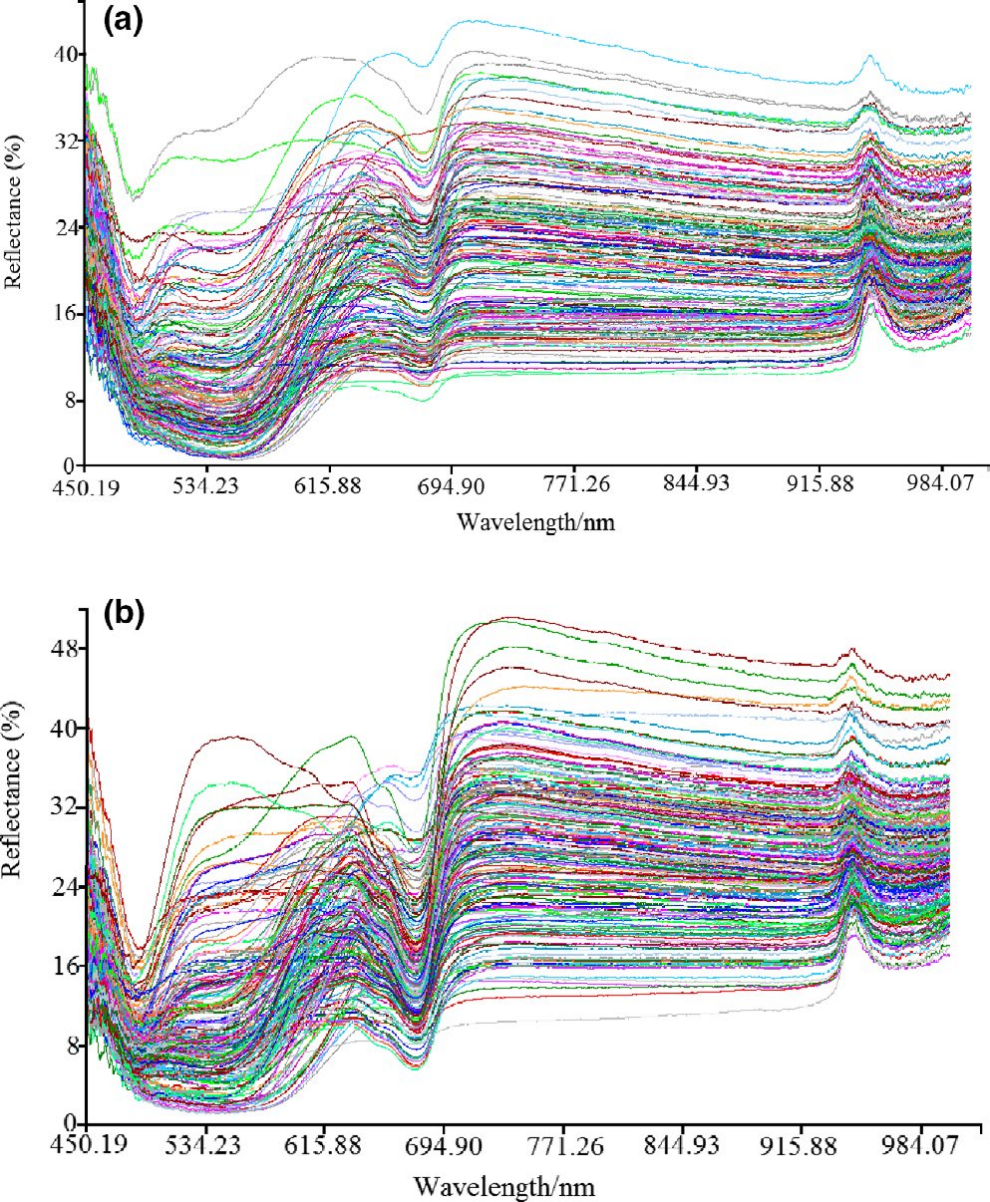
Spectral reflectance curves of “Fuji” and “Qinguan” apple samples

To avoid noise interference in the head and tail of the spectral reflectance curve and improve accuracy and robustness of the model, the range of wavelength was selected from 450 nm to 1,000 nm. Figure 2a,b display the spectral reflectance curves of “Fuji” and “Qinguan” samples. Owing to individual variation, spectral reflectance curve of each apple sample presented differently, meaning the reflectance curves were scattered. However, on the whole, there was a trough near 670 nm and a crest near 950 nm. The trough was the absorption peak, and the crest was the reflectance peak. To further quantitatively analyze the apples' crispness, chemometrics were used to mine effective optical fiber spectral information.

### 3.2 Spectral preprocessing

The NIPLS algorithm was used to establish a quantitative prediction model that related apples' crispness with the optical fiber spectral data (reflectance). To improve prediction accuracy, the raw spectra (RS) data need to be preprocessed before establishing the model. The spectral preprocessing techniques include Savitzky–Golay smoothing (S‐G smoothing, where the smooth points of left and right were 20), first derivation (1‐Der, where the smooth points of left and right were 25), second derivation (2‐Der, where the smooth points of left and right were 25), and standard normal variate (SNV). Table 1 shows the modeling results of “Fuji” apple and “Qinguan” apples following spectral preprocessing.

**Table 1:** Results of modeling with preprocessing methods

Apple cultivar	Pretreatment methods	*N*	R_C_^2^	RMSE_C_/g	R_P_^2^	RMSE_P_/g
Fuji	RS	2	0.8939	125.43	0.9206	166.87
	S‐G	2	0.8940	125.42	0.9205	166.87
	1‐Der	4	0.8459	151.21	0.7526	294.43
	2‐Der	5	0.8659	141.06	0.6618	344.28
	SNV	9	0.8927	126.17	0.5709	387.78
Qinguan	RS	2	0.9406	140.82	0.9587	174.13
	S‐G	2	0.9406	140.83	0.9587	174.14
	1‐Der	4	0.8887	192.73	0.8144	369.21
	2‐Der	5	0.7955	261.23	0.5546	571.94
	SNV	9	0.7977	259.80	0.5682	563.10

Abbreviations: 1‐Der, first derivation; 2‐Der, second derivation; *N*, number of principal components; R_C_, correlation coefficient of calibration set; RMSE_C_, root mean square error of the calibration set (g); RMSE_P_, root mean square error of the prediction set (g); R_P_, correlation coefficient of prediction set; RS, raw spectra; S‐G, Savitzky–Golay smoothing; SNV, standard normal variate.

As can be seen in Table 1, the established PLS model based on S‐G smoothing preprocessing was better at predicting crispness of “Fuji” apples, and the established PLS model based on raw spectra (RS) was better at predicting crispness of “Qinguan” apples.

After spectral preprocessing, the calibration set and prediction set were divided according to a ratio of 3:1 using the Kennard–stone (KS) algorithm in MATLAB (matric laboratory). For “Fuji” apple samples, the number of calibration set and prediction set was 147 and 50, respectively. For “Qinguan” apple samples, the number of calibration set and prediction set was 164 and 55, respectively.

### 3.3 Effective wavelengths

To simplify model and improve efficiency of modeling, selection of effective wavelength information was taken into consideration. To facilitate the development of instrument, useless spectral information was discarded. The SPA algorithm and the x‐LW method were used to select effective wavelengths. The effective wavelengths selected using SPA algorithm for “Fuji” apple samples were 450.51 nm, 476.80 nm, 677.75 nm, and 750.72 nm. The selected effective wavelengths via SPA algorithm for “Qinguan” apple samples were 460.01 nm, 463.21 nm, 507.55 nm, and 725.19 nm. Compared with the full wavelength (FW), SPA algorithm considerably reduced the size of wavelengths.

**Figure 3:**
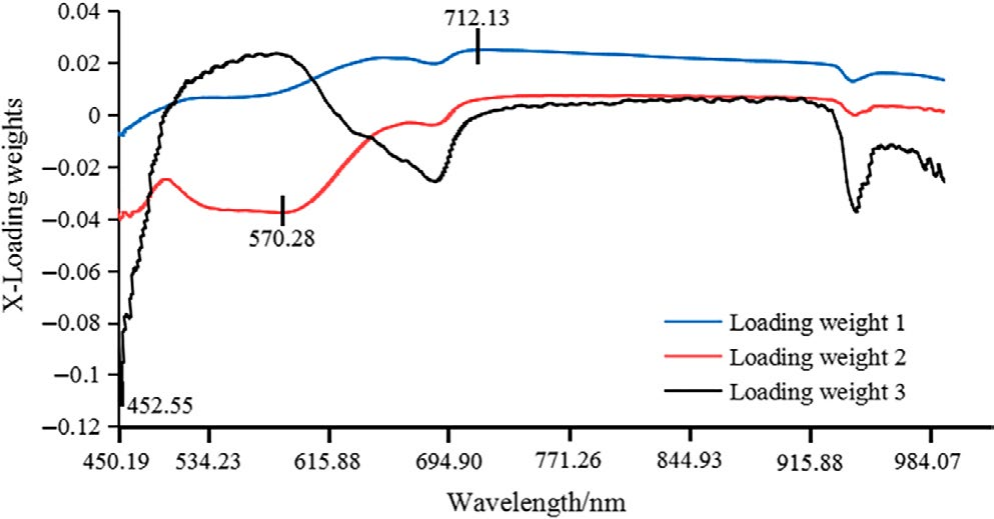
Effective wavelengths selected by x‐LW based on “Fuji” apple samples

**Figure 4:**
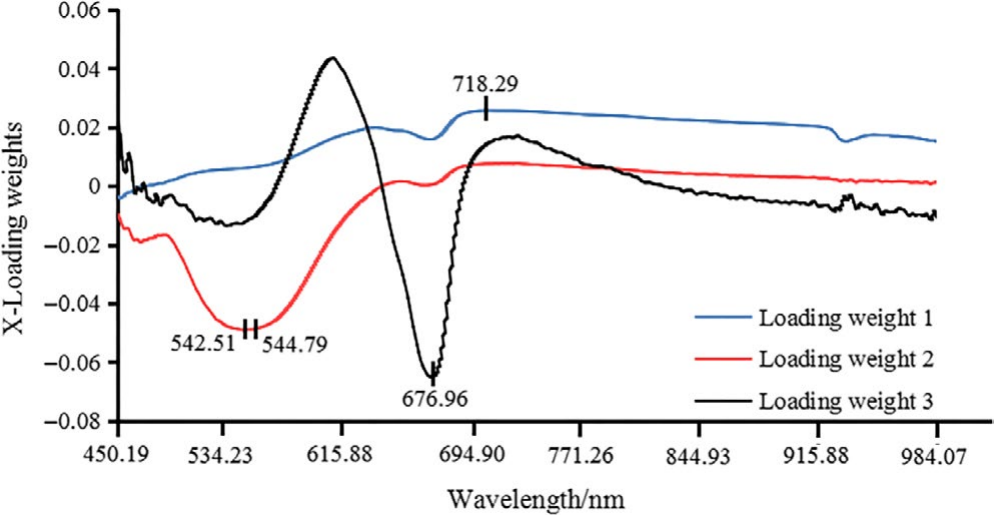
Effective wavelengths selected by x‐LW based on “Qinguan” apple samples

The x‐LW method was dependent on PLS model. The PLS model was developed based on the full wavelength and was able to obtain the best implicit variables, of which there were four. Figures 3 and 4 show the load distribution for the first three implicit variables based on “Fuji” apple samples and “Qinguan” apple samples. The maximum absolute value of load coefficient for each implicit variable was obtained. The wavelength corresponding to the maximum absolute value was determined as the effective wavelength for each implicit variable. Figure 3 presents the selected effective wavelengths obtained via x‐LW method for “Fuji” apple samples, which were 452.55 nm, 570.28 nm, and 712.13 nm. Figure 4 presents the selected effective wavelengths obtained via x‐LW method for “Qinguan” apple samples, which were 542.51 nm, 544.79 nm, 676.96 nm, and 718.29 nm. Quantitative prediction models were developed via the newly selected characteristic variables.

### 3.4 Establishment and analysis of quantitative models

The NIPLS algorithm was used to develop quantitative prediction models. Quantitative prediction models were based on the full wavelength, allowing us to obtain the selected effective wavelengths Table 2 shows the results of different models based on “Fuji” and “Qinguan” apple samples. Throughout the PLS modeling process using the software Unscrambler X 10.1, spectral data were viewed as independent variables, and crispness values were viewed as dependent variables. The optimal principal component, prediction residual error sum of squares (PRESS), and standard error of cross‐validation (SECV) were important model parameters, which were able to be determined via cross‐validation. The values of PRESS and SECV were calculated under the condition of the different number of components. The maximum component was set to an initial value of 15 (generally from 10 to 20) and adjusted according to the modeling result. The optimal principal component was obtained with the component having the smallest value of PRESS and SECV. The mean center data and identify outliers were chosen (and the outliers potentially removed), which can help optimize the model. Cross‐validation was regarded as the validation method. The PLS model was able to be obtained after setting the necessary model parameters, and the best model effect was determined according to the changeable number of components. The different number of components corresponded with different model effects. Therefore, the PLS model was able to be optimized by changing the number of components and model evaluation methods. Additionally, it was also effective to optimize the model by changing the method of sample division, which can involve changing the ratio of calibration set and prediction set.

**Table 2:** Differentiated results of different models for “Fuji” and “Qinguan” apple cultivars

Model	Vn	Calibration set			Prediction set		
		Sn	RMSE_C_/g	*R*‐square	Sn	RMSE_P_/g	*R*‐square
Fuji							
FW‐SG‐PLS	2,896	147	125.42	.8940	50	166.87	.9205
SPA‐PLS	4	147	145.02	.8756	50	114.97	.9131
x‐LW‐PLS	3	147	129.36	.8872	50	193.28	.8934
Qinguan							
FW‐RS‐PLS	2,896	164	140.83	.9406	55	174.14	.9587
SPA‐PLS	4	164	103.77	.9779	55	189.95	.8629
x‐LW‐PLS	4	164	135.80	.9447	55	162.32	.9641

Abbreviations: RMSE_C_, root mean square error of the calibration set (g); RMSE_P_, root mean square error of the prediction set (g); Sn, sample number; Vn, variables number.

To simplify the model, a quantitative prediction model was redeveloped based on effective wavelengths which were previously selected by SPA algorithm and x‐LW method. For “Fuji” apple samples, four effective wavelengths were selected via SPA algorithm and three effective wavelengths were selected via x‐LW method. For “Qinguan” apple samples, four effective wavelengths were selected via SPA algorithm and x‐LW method, respectively. Overall, the effects of SPA‐PLS model and x‐LW‐PLS model were very good. The differentiated results of modeling for “Fuji” and “Qinguan” apple samples are shown in Table 2.

The SPA‐PLS model and the x‐LW‐PLS model used for “Fuji” apple samples were expressed as formula (1) and formula (2), respectively. In practice, formula (1) was tend to applied to “Fuji” apple samples, because SPA‐PLS model achieved better prediction accuracy than x‐LW‐PLS.

(1) $$\begin{array}{c} C_{\text{Fuji}}=3.5527X_{450\text{.41}}-12.2116X_{476.80}\\ +11.1806X_{677\text{.75}}+58.0022X_{750\text{.72}}+1494.77 \end{array}$$

(2) $$\begin{array}{c} C_{\text{Fuji}}=-9.3615X_{452\text{.55}}-0.0747X_{570\text{.28}}\\ +57.5901X_{712\text{.13}}+1864.9304 \end{array}$$

*C*_Fuji_ = crispness of “Fuji” apple; *X*_450.41_, *X*_452.55_, *X*_476.80_, *X*_570.28_, *X*_677.75_, *X*_712.13_, and *X*_750.72_ indicate spectral reflectance of different effective wavelengths, respectively.

The SPA‐PLS model and the x‐LW‐PLS model for “Qinguan” apple samples were expressed as formula (3) and formula (4), respectively. In practice, formula 4 was generally applied to “Qinguan” apple samples as the x‐LW‐PLS model was able to achieve better prediction results for this cultivar than SPA‐PLS.

(3) $$\begin{array}{c} C_{\text{Qinguan}}=8.8838X_{460\text{.01}}+2.4230X_{463\text{.21}}\\ -9.0757X_{507\text{.55}}+92.8994X_{725\text{.19}}-241.4977 \end{array}$$

(4) $$\begin{array}{c} C_{\text{Qinguan}}=-2.1648X_{542\text{.51}}-1.4604X_{544\text{.79}}\\ -2.1189X_{676\text{.96}}+89.7941X_{718\text{.29}}+27.950 \end{array}$$

*C*_Qinguan_ = crispness of “Qinguan” apple; *X*_460.01_, *X*_463.21_, *X*_507.55_, *X*_542.51_, *X*_544.79_, *X*_676.96_, *X*_718.29_, and *X*_725.19_ indicate spectral reflectance of different effective wavelengths, respectively.

Based on the comparison of above three models, optimal model and optimal selection method of effective wavelengths for “Fuji” and “Qinguan” apple samples were obtained. For “Fuji” apple samples, the modeling and prediction accuracy of FW‐SG‐PLS model were the best. However, to facilitate the development of a crispness detection instrument, it is necessary to choose the most effective wavelengths and develop a superior model. As far as prediction accuracy is concerned, according to Table 2, SPA‐PLS model was better than x‐LW‐PLS model when applied to “Fuji” apples because it possessed larger *R* ^2^ value and smaller RMSE value. For “Qinguan” apple samples, modeling and prediction accuracy of x‐LW‐PLS model were superior due to larger *R* ^2^ value and smaller RMSE value. Therefore, in order to improve the efficiency of modeling and facilitate the development of a crispness detection instrument, SPA algorithm was used to select effective wavelengths for “Fuji” apple samples, and x‐LW method was used to select effective wavelengths for “Qinguan” apple samples.

### 3.5 Model prediction based on artificial neural network method (ANN)

SPSS software was used to build the quantitative prediction model via ANN, RBNN, and MLPNN. The reflectance of selected effective wavelengths was regarded as input variables, and crispness values of apple samples were treated as output variable. Training set and test set were divided according to a ratio of 7:3, and the abnormal samples were eliminated.

Table 3 shows model establishment results of “Fuji” apple samples based on RBNN and MLPNN methods. Throughout the ANN modeling process using the software SPSS, hidden layers and activation function were the main model parameters to optimize the ANN model. The better prediction model could be obtained by changing the number of hidden layers and activation function. It was also effective to optimize the model by changing the number of hidden layer units. The determination coefficient (*R* ^2^), the sum of square deviation, and relative deviation were used to evaluate the ANN model; the lower the sum of square deviation and relative deviation are, the more effective the model will be. Changing the ratio of training set and test set was also effective to optimize the model. As for the RBNN model, improved prediction results were obtained when activation functions of hidden layer were softmax function and exponential function, and activation function of output layer was identity function. In addition, different activation functions of hidden layer corresponded to different units; the softmax and exponential functions corresponded to nine units and ten units, respectively. As for the MLPNN model, improved prediction results were obtained when activation function of hidden layer was hyperbolic tangent, and activation function of output layer was identity. At the same time, the units of layer 1 and layer 2 were 20 and 15, respectively.

**Table 3:** Model establishment of “Fuji” apple samples based on MLPNN and RBNN

	Input layer	Hidden layer			Output layer	
	Vn	Layer 1	Layer 2	Af	Vn	Af
		units				
MLPNN	4	20	15	Hyperbolic tangent	1	Identity
RBNN	4	9		Softmax	1	Identity
	4	10		Exponential		

Abbreviations: Af, activation function; MLPNN, multilayer perceptron neural network; RBNN, radial basis neural networks; Vn, variables number.

**Figure 5:**
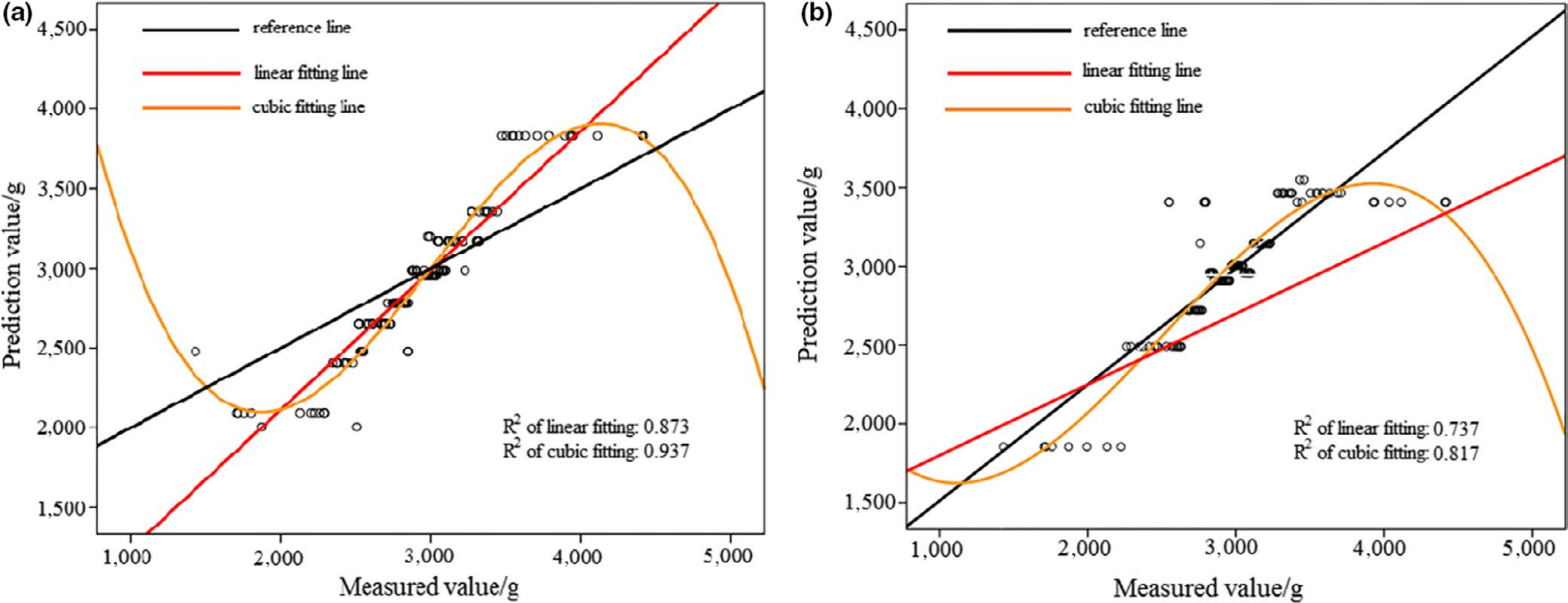
Prediction result of “Fuji” apple samples based on RBNN

**Figure 6:**
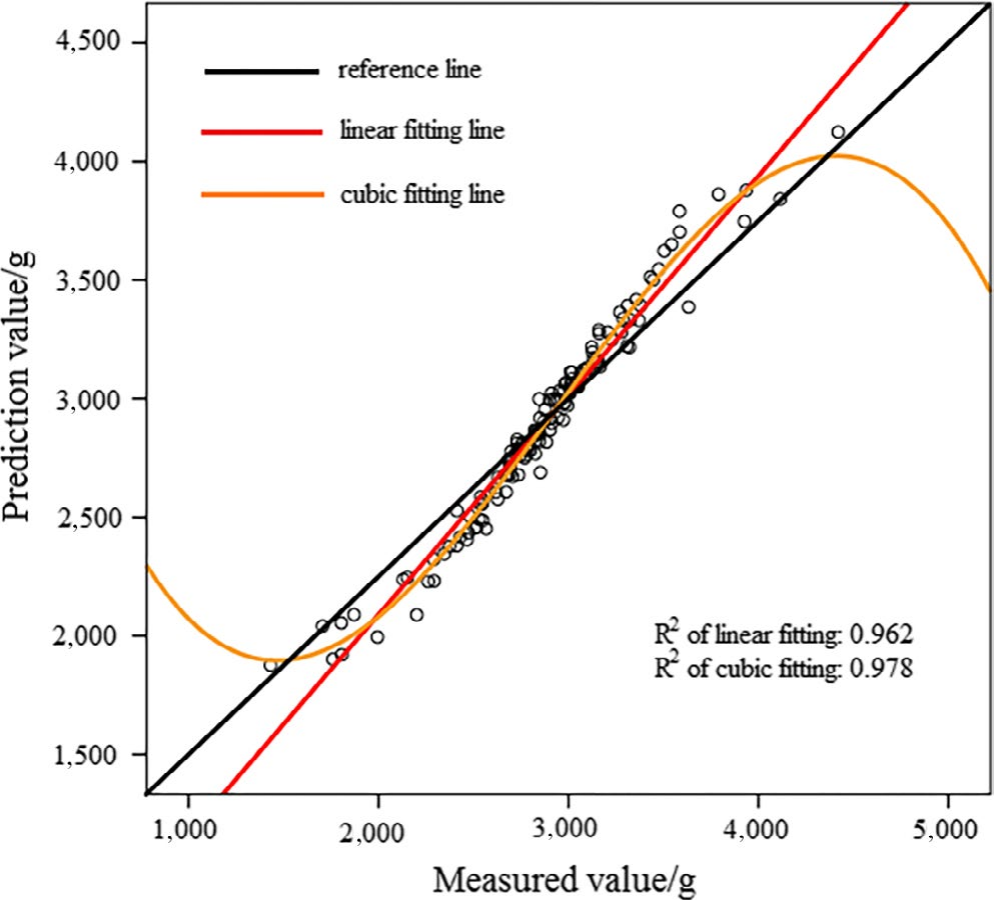
Prediction result of “Fuji” apple samples based on MLPNN

In Figure 5a, the activation function of hidden layer was exponential. In Figure 5b, the activation function of hidden layer was softmax. According to Figures 5 and 6, prediction results of MLPNN model were better than that of RBNN model. Nonlinear fitting was also more effective than linear fitting. Table 5 shows deviations of RBNN and MLPNN models; the MLPNN model possessed a smaller sum of square deviation and relative deviation for “Fuji” apple samples, indicating that MLPNN model was more accurate and stable for “Fuji” apple samples.

As with “Fuji,” the prediction model for “Qinguan” apple samples based on RBNN and MLPNN methods was also established, and the results are presented in Table 4. As can be observed, one RBNN model and two MLPNN models were developed according to the different activation function of hidden layer.

**Table 4:** Model establishment for “Qinguan” apple samples based on RBNN and MLPNN

	Input layer	Hidden layer		Output layer	
	Vn	units	Af	Vn	Af
RBNN	4	9	Exponential	1	Identity
MLPNN	4	20	Hyperbolic tangent Sigmoid	1	Identity

Abbreviations: Af, activation function; MLPNN, multilayer perceptron neural network; RBNN, radial basis neural networks; Vn, variables number.

**Figure 7:**
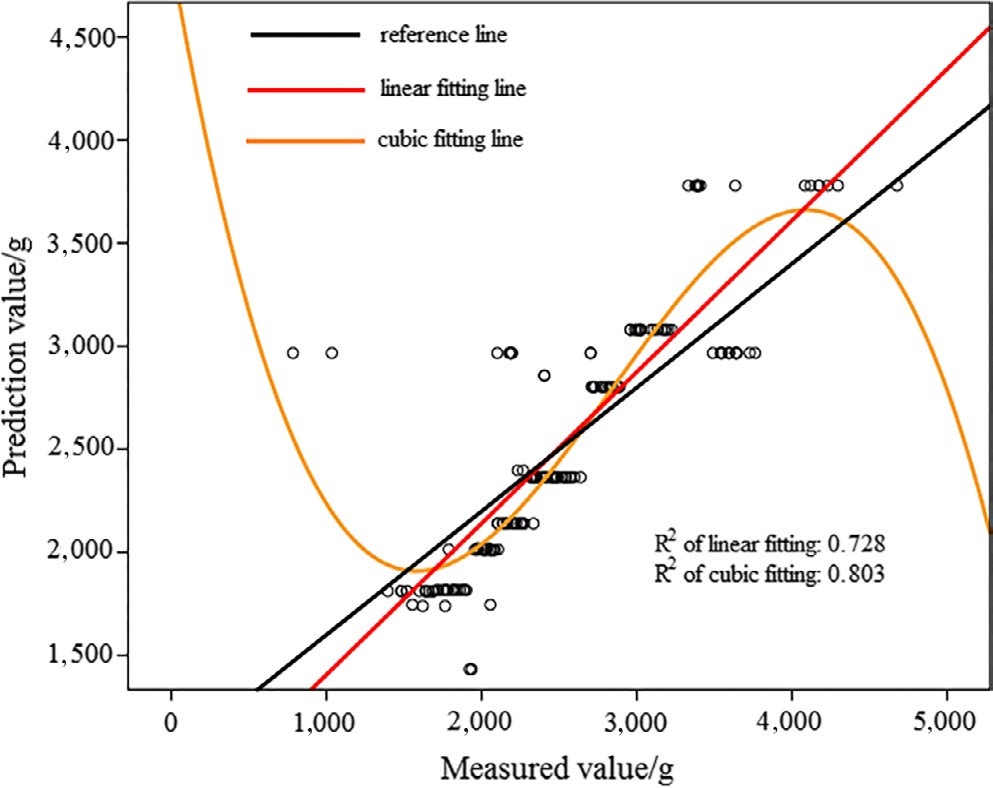
Prediction result of “Qinguan” apple samples based on RBNN

**Figure 8:**
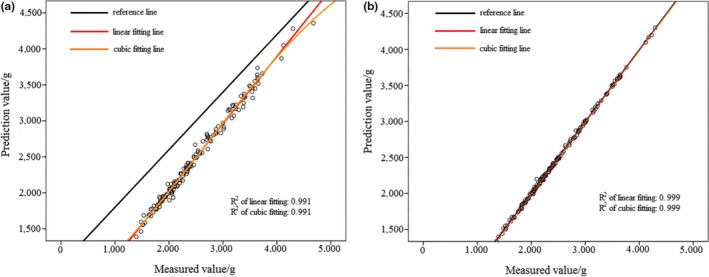
Prediction result of “Qinguan” apple samples based on MLPNN

In Figure 8a, activation function of hidden layer was sigmoid. In Figure 8b), activation function of hidden layer was hyperbolic tangent. Based on Figures 7 and 8, it can be observed that prediction result of MLPNN model was better than that of RBNN model for “Qinguan” apple samples. According to Figure 8, when the activation function was hyperbolic tangent, the MLPNN model achieved better prediction results because of the larger *R* ^2^ value. Table 5 displays the sum of square deviation and relative deviation of RBNN and MLPNN models, with the MLPNN model having a smaller sum of square deviation and relative deviation for “Qinguan” apple samples. This result confirmed that MLPNN model was more accurate and stable for “Qinguan” apple samples.

**Table 5:** Deviations of RBNN and MLPNN models for “Fuji” and “Qinguan” apple samples

Model	Units		Training set		Test set	
			Ssd	Rd	Ssd	Rd
Fuji						
RBNN	9		17.764	0.263	0.066	0.349
	10		8.271	0.127	0.033	0.106
MLPNN	Layer 1	20	2.953	0.043	0.001	0.009
	Layer 2	15				
Qinguan						
RBNN	9		21.549	0.266	0.755	10.090
MLPNN	20(Hyperbolic tangent)		0.054	0.001	0.013	0.070
	20(Sigmoid)		0.942	0.013	0.009	0.040

Abbreviations: MLPNN, multilayer perceptron neural network; RBNN, radial basis neural networks; Rd, relative deviation; Ssd, sum of square deviation.

Using the above comparison, it can be seen that MLPNN model was the best regardless of whether it was applied to “Fuji” or “Qinguan” apple samples. On the whole, cubic fitting was more effective than linear fitting. This also indicated the selected effective wavelengths were valid and reliable. According to prediction results and the *R* ^2^ value, apple crispness could be predicted accurately and validly using the MLPNN model.

Above all, modeling procedures were obtained via PLS and ANN modeling methods, with the most effective models for “Fuji” and “Qinguan” apple samples obtained via a respective comparison of modeling results. Therefore, it is feasible to predict apple crispness using the PLS and ANN modeling methods. The established models were able to be transferred into program of Python and then inserted into Raspberry Pi in order to control spectrometer or light‐emitting diode (LED), thereby detecting the apples' crispness. The LED based on effective wavelengths was also used to be used to collect spectral information of apples' crispness. Following this, apple crispness could be obtained via the established model. Therefore, a method for nondestructively detecting apple crispness was presented and it can be applied in a real‐world scenario. In addition, selecting effective wavelengths were able to facilitate the development of a crispness detection instruments.

## 4 CONCLUSIONS

The nondestructive detection of apple crispness for both “Fuji” and “Qinguan” apple cultivars was successfully accomplished. In order to detect apple crispness in a nondestructive manner, a technique that combined spectroscopy with PLS and ANN modeling methods was proposed.

1. Based on the comparison of x‐LW method and SPA algorithm, selected effective wavelengths for “Fuji” apple samples were 450.41 nm, 476.80 nm, 677.75 nm, and 750.72 nm. For “Qinguan” apple samples, they were 542.51 nm, 544.79 nm, 676.96 nm, and 718.29 nm. The selected effective wavelengths were able to potentially include the spectral information regarding apples' crispness. Furthermore, the effective wavelengths were able to improve modeling efficiency and facilitate the development of a practical crispness detection instrument.
2. The prediction accuracy of the PLS model for “Fuji” and “Qinguan” apple samples based on the selected effective wavelengths achieved 91.31% and 96.41%, respectively. To better predict apple crispness, a range of ANN models was developed, with the MLPNN model achieving better nonlinear prediction results via a comparison of RBNN and MLPNN models. The prediction accuracy of MLPNN model for “Fuji” and “Qinguan” apple samples based on the selected effective wavelengths reached 97.8% and 99.9%, respectively. By comparing the PLS model with ANN models, the effects of ANN models were markedly superior to that of the PLS model. Therefore, to nondestructively detect apple crispness, an algorithm (such as artificial neutral networks) should be adopted as a modeling method due to its superior prediction accuracy.
3. Based on the above, by validating and optimizing the prediction model, SPA algorithm was used to select effective wavelengths for “Fuji” apple cultivar and x‐LW method was used to select effective wavelengths for “Qinguan” apple cultivar. The established effective wavelength‐based ANN model was shown to be both reliable and accurate. Therefore, it is feasible to nondestructively detect apple crispness via optical fiber spectroscopy, combined with both effective wavelengths and the ANN modeling method.

## CONFLICT OF INTEREST

The authors declare that they have no conflict of interest to this research.

## ETHICAL APPROVAL

Ethical Review: This study does not involve any human or animal testing; this study was approved by the Institutional Review Board of Northwest A&F University; this study conforms to the Declaration of Helsinki, US.

## INFORMED CONSENT

Written informed consent was obtained from all study participants.
